# Construction of 3D hierarchical tissue platforms for modeling diabetes

**DOI:** 10.1063/5.0055128

**Published:** 2021-10-20

**Authors:** Myungji Kim, Jinah Jang

**Affiliations:** 1 School of Interdisciplinary Bioscience and Bioengineering, POSTECH, 77 Cheongam-ro, Namgu, Pohang, Kyungbuk, 37673, Republic of Korea; 2 Department of Convergence IT Engineering, POSTECH, 77 Cheongam-ro, Namgu, Pohang, Kyungbuk, 37673, Republic of Korea; 3 Department of Mechanical Engineering, POSTECH, 77 Cheongam-ro, Namgu, Pohang, Kyungbuk, 37673, Republic of Korea; 4 Institute of Convergence Science, Yonsei University, 50 Yonsei-ro, Seodaemun-gu, Seoul, 03722, Republic of Korea

## Abstract

Diabetes mellitus (DM) is one of the most serious systemic diseases worldwide, and the majority of DM patients face severe complications. However, many of underlying disease mechanisms related to these complications are difficult to understand with the use of currently available animal models. With the urgent need to fundamentally understand DM pathology, a variety of 3D biomimetic platforms have been generated by the convergence of biofabrication and tissue engineering strategies for the potent drug screening platform of pre-clinical research. Here, we suggest key requirements for the fabrication of physiomimetic tissue models in terms of recapitulating the cellular organization, creating native 3D microenvironmental niches for targeted tissue using biomaterials, and applying biofabrication technologies to implement tissue-specific geometries. We also provide an overview of various *in vitro* DM models, from a cellular level to complex living systems, which have been developed using various bioengineering approaches. Moreover, we aim to discuss the roadblocks facing *in vitro* tissue models and end with an outlook for future DM research.

## I. INTRODUCTION

As a global epidemic, diabetes mellitus (DM) is a chronic disorder characterized by the dysfunction of beta cells in the pancreatic islets, leading to impaired glucose metabolism and severe episodes of complications (e.g., diabetic cardiomyopathy, retinopathy, and nephropathy). This disease represents a growing financial burden for public health systems. In 2019, approximately 463 million people suffered from DM worldwide, and the International Diabetes Federation estimates that around 630 million individuals will suffer from diabetes by 2045.^1^ Type 1 (T1DM) and type 2 (T2DM) are the most common forms of DM, and both are characterized by complex pathophysiology. T1DM is classified as an autoimmune disease triggered by a range of genetic and environmental factors that lead to the destruction of insulin-secreting beta cells. T2DM, which accounts for over 90% of DM patients, occurs when beta cells are unable to secrete sufficient insulin to satisfy the physiological demands of insulin-responsive organs such as the liver and skeletal muscle.^2^ T2DM is a multifactorial disease, but it is clear that obesity-induced insulin resistance is the most prominent cause of beta cell exhaustion leading to relative insulin deficiency.^3^

The majority of DM patients face severe long-term complications from the disease. The main cause of diabetic complications is systemic damage in noticeably impaired macro- and microvasculatures induced by hemodynamic and metabolic changes. It has been shown that patients with macrovascular complications have a higher risk of atherosclerosis resulted in myocardial infarction (MI) than those who do not have complications.^4^ In particular, diabetic cardiomyopathy (DCM), which is characterized by left ventricular dysfunction and abnormal myocardial insulin signaling in the absence of coronary artery disease and hypertension, is increasingly regarded as a serious complication among T2DM patients.^5–7^ Microvascular complications are strongly associated with chronic hyperglycemia including diabetic retinopathy, nephropathy, and peripheral neuropathy, and these are recognized as the main causes of blindness, kidney failure, and damage to the lower limbs, respectively.^4^ Although the prevalence of diabetes-induced vascular manifestations is continuously rising, many of signaling pathways related to these complications remain poorly understood.

To examine the pathogenesis and underlying mechanisms of DM, many researchers have developed a wide range of experimental animal models. For instance, non-obese diabetic mice, Akita mice, BioBreeding rats, and LEW.1AR1 rats are important models for studying T1DM. Numerous genes, including major histocompatibility complexes and cytotoxic T-lymphocyte-associated protein 4 discovered in NOD mice, have been proven to contribute to T1DM susceptibility in humans.^8^ In addition, streptozotocin (STZ) and alloxan are cytotoxic glucose analog used in chemically induced hyperglycemia and hypoinsulinaemia.^9,10^ As single injection of STZ, which is less toxic than alloxan, can readily induce T1DM in rodents, STZ-involved diabetic animals have been considered as stable, cost-effective, and time-saving platforms for investigating pharmacological reagents.^11^ Studies identifying the pathogenesis of T2DM have commonly been conducted on mice and rats fed a high fat diet, Zucker Diabetic Fatty rats, and Goto-Kakizaki (GK) rats. GK rats, in particular, have allowed researchers to better understand the complex association between beta cell failure and T2DM as a non-overweight T2DM model due to the depletion of the beta cell mass.^12^ Although animal models for DM have greatly contributed to understanding of the pathological mechanisms underlying DM by embodying key characteristics of the metabolic disorder, animal models cannot fully represent the pathology of the disease as found in humans. For example, humans and rodents have different islet characteristics (e.g., their structure, cell composition, and metabolic process) and immune responses related to the pathophysiology of T1DM.^3,13,14^ Therefore, the translation of results derived from animal models to humans in preclinical assessments that test the efficacy and safety of novel treatment strategies remains controversial due to their limitations in recapitulating human-specific physiology, metabolism, and genetics.^15^

**FIG. 1.**
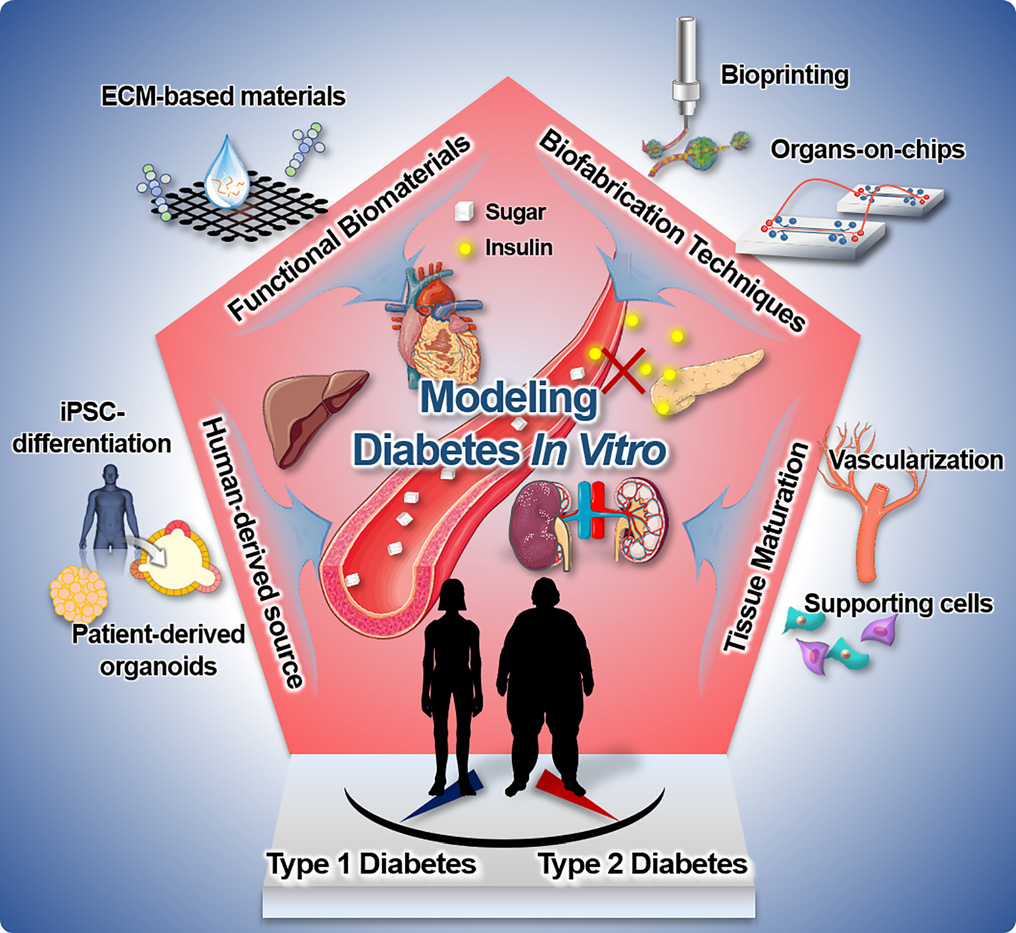
An overview of various approaches for DM modeling *in vitro*.

With the tremendously increasing demand for the systemic prediction of human relevant *in vivo* responses, various 3D *in vitro* disease models based on tissue engineering approaches have been developed as a pharmaceutical testing platform for future clinical studies. Traditional two-dimensional (2D) models are able to replicate certain characteristics of DM pathophysiology *in vitro*, but fundamental translation to *in vivo* human physiology requires a complex 3D native architecture.^16,17^ In order to overcome the limitation, the use of extracellular matrix-derived materials has emerged as a potential strategy for the creation of native 3D microenvironmental niches for targeted tissue. Furthermore, substantial progress has been made in the development of bioengineering strategies such as biofabrication, microfluidics, and stem cell engineering that can provide various biomimetic cues and advanced functions to the *in vitro* disease models (Fig. 1).

In this review, we describe the essential requirements for the production of functional *in vitro* models in terms of optimizing the cellular composition, recapitulating *in vivo*-like 3D microenvironments using biomaterials, and employing biofabrication technologies to manufacture the biomimetic human tissue architecture.^18,19^ We begin by describing a variety of *in vitro* DM models, from cells to complex living systems, that have been developed using diverse engineering strategies. Furthermore, we address the major challenges facing *in vitro* tissue modeling and, finally, discuss future directions for DM research.

## II. MODELING DM AT THE ORGANOTYPIC CELLULAR LEVEL

Various cell sources, from primary to stem cell-derived cells, have been employed in *in vitro* DM modeling. Isolated human and animal pancreatic islets have been frequently used as cell sources to identify disease mechanisms and validate the therapeutic effects of a variety of treatments in diabetes research.^20^ Human islets can be considered the gold standard in terms of understanding the uniqueness of human islet biology, but the limited supply of human islets and the high costs associated with the isolation process remain significant limitations.^21^ Accordingly, many studies have been conducted using cell lines as an alternative source for DM studies.^22^ Jiang *et al.* demonstrated the role of metformin, the first-line oral medication for T2DM, in rodent-derived MIN6 cell lines.^23^ They induced apoptosis in MIN6 cells using palmitic acid to simulate the conditions of chronic free fatty acid (FFA) exposure and observed the functional role of metformin. It was found that the activation of autophagy via AMP-activated protein kinase signaling participated in protecting beta cells from FFA-induced apoptosis. Their findings indicated that a pancreatic beta cell line can serve as a useful tool for the study of the complex nature of drug action against DM *in vitro*.

Although identifying the molecular mechanism of diabetogenic drugs in commonly used animal cell lines can assist in determining the appropriate use of anti-diabetic drugs, a physiologically human tissue-relevant platform based on human-derived cells is required for further clinical insights. Recently, Leite *et al.* successfully implemented autoimmune human T1DM model *in vitro* using patient-derived human induced pluripotent stem cells (hiPSCs) with autologous immune cells.^24^ The developed disease model was combined with iPSC-derived pancreatic endocrine cells (e.g., beta cells and alpha cells) and peripheral blood mononuclear cells (PMBCs) isolated from patients to simulate the immune interactions observed in T1DM pathogenesis following thapsigagin treatment, which triggers ER stress. They found that the survival rate of beta cells was lower than glucagon-secreting alpha cells when co-cultured with autologous PBMCs, indicating the preferential T cell-mediated destruction of beta cells. Their model identified the key components associated with the induction of immunopathogenesis of T1DM and confirmed the feasibility of predicting the outcomes of patient-specific autoimmune responses *in vitro*.

**FIG. 2.**
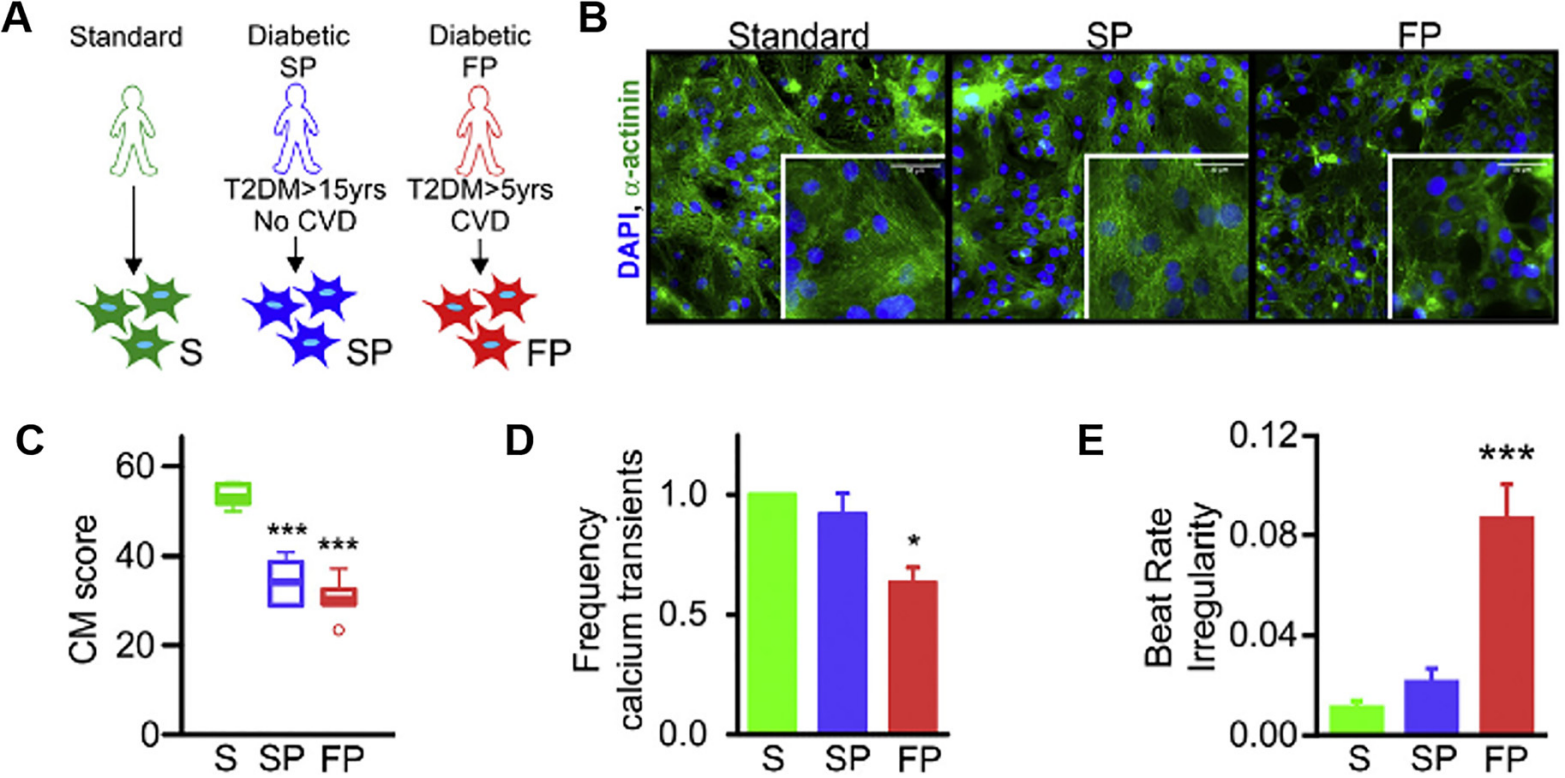
DM patient specific CMs revealed DCM phenotypes without external diabetic stimulation. (a) Schematic to show characteristics of DM patients. (b) Immunofluorescent images of three types of CM. Scale bar, 30 *μ*m. (c) CM score, which indicates quantified expression of a-actinin, in FP CMs showed lower level than standard and SP CMs. (d) Calcium-transient frequency of each type of CMs. (e) Beat-rate irregularity of FP CMs has significantly increased. Error bars: ∗p < 0.05, ∗∗p < 0.01, and ∗∗∗p < 0.001. Reprinted with permission from Drawnel *et al.*, Cell Rep. **9**, 810–820 (2014). Copyright 2014 Elsevier.^25^

Furthermore, Drawnel *et al.* established the foundation for the modeling of diabetic macrovascular complications connected to the primary causes of death in T2DM using cardiomyocytes (CMs) derived from diabetic patient-specific iPSCs.^25^ In this study, they highlighted the importance of cellular maturation for the recapitulation of DCM, which is a disease of adult CMs and characterized the properties of mature iPSC-derived CMs after promoting metabolic patterns of adult cardiac activity. They subsequently applied a diabetic stimulus to the CMs using a high concentration of glucose and hormonal mediators of DM (e.g., endothelin 1 and cortisol), leading to the disruption of the sarcomere structure, lipid accumulation, and peroxidation. Gene set enrichment analysis results revealed the suppression of genes related to the production of proteins (e.g., mitosis, DNA repair, and protein translation) after DM treatment, which means that their model reflected the transition to a diseased state.^26^ Interestingly, cardiomyopathic phenotypes, such as fast progression (FP) and slow progression (SP), were also observed in diabetic patient-specific CM models in the absence of diabetic manipulation **(**Fig. 2). Moreover, they validated that patient-specific DCM displayed clinically correlated results through the phenotypic drug screening system. This type of research, based on patient-derived cell sources, had a significant clinical impact on optimizing the specificity and efficacy of therapeutic molecules for the diabetic patients.

Organoids, also known as a stem cell-derived 3D mini-organ, have been utilized for the valuable tools for investigating functional hallmarks of native organ beyond 2D stem cell models.^27,28^ Organoids replicate both the healthy and disease states of human organs with the intrinsic ability to reproduce the multicellular repertoire of human physiology *in vitro*.^29,30^ At present, research works on endocrine pancreatic organoids have mainly focused on the generation of beta cells for the treatment of T1DM following transplantation.^31^ Pancreatic islets are composed of multiple types of hormonal cells that affect dysregulation observed in diabetes. In this regard, Peterson *et al.* argued that the generation of stem cell-derived glucagon-producing alpha cells, which play a role in raising glucose levels by regulating glycogenolysis in the liver for use in islet organoids, can accelerate the exploration of diabetic disease progression.^32^ Obviously, the research involving recapitulation of the unique heterogenicity of the target tissues *in vitro* could advance efforts to discover the various pathological responses of diabetic organs. As an intriguing example, a recent DM study by Wimmer *et al.* suggested that organoids can be used as a reliable platform to identify largely unknown pathways related to DM complication. They generated a human diabetic vasculopathy model relative to microvascular complications via organoids technology to probe the 3D physiological features of the entire human blood vessels under hyperglycemia conditions.^33^ They differentiated hiPSCs into vascular organoids to form lumenized 3D capillary networks with endothelial cells (ECs), pericytes, mesenchymal stem-like cells, and hematopoietic cells. The vascular organoids were exposed to a diabetic medium comprised of high glucose, human TNF, and IL-6 to emulate the hyperglycemic and inflammatory situation in DM. Significantly thicker vascular basement membranes and a lower capillary density, both of which are representative of the dermal microvasculature in T2DM patients, were identified in the diabetic lumina of the 3D human blood vessel organoids. The blood vessel organoids contributed to the discovery of major mediators of vessel pathology, including NOTCH3 and DLL4, thus demonstrating their applicability in the development of drugs that ameliorate the impairment of the microvascular system due to DM.^34^

## III. MODELING DM USING HIERARCHICAL 3D CONSTRUCTS

Intense studies have been conducted on DM therapeutics with encapsulation of pancreatic islets in natural biomaterials (e.g., alginate, silk, and collagen) to sustain the 3D structural hierarchy and functionality of the islets.^35–39^ Recently, the definition of implementation of 3D biomimetic environment has expanded as a meaning of creating a realistic living system that can enhance the maturity of the cells by providing inherent tissue-specific environmental cues beyond simply maintaining the viability of the encapsulated cells.^40–42^ In this section, the need of 3D physiomimetic microenvironmental cues to create a superior 3D human DM model *in vitro* will be addressed.

### A. Modeling DM with cell-laden 3D hydrogel

**FIG. 3.**
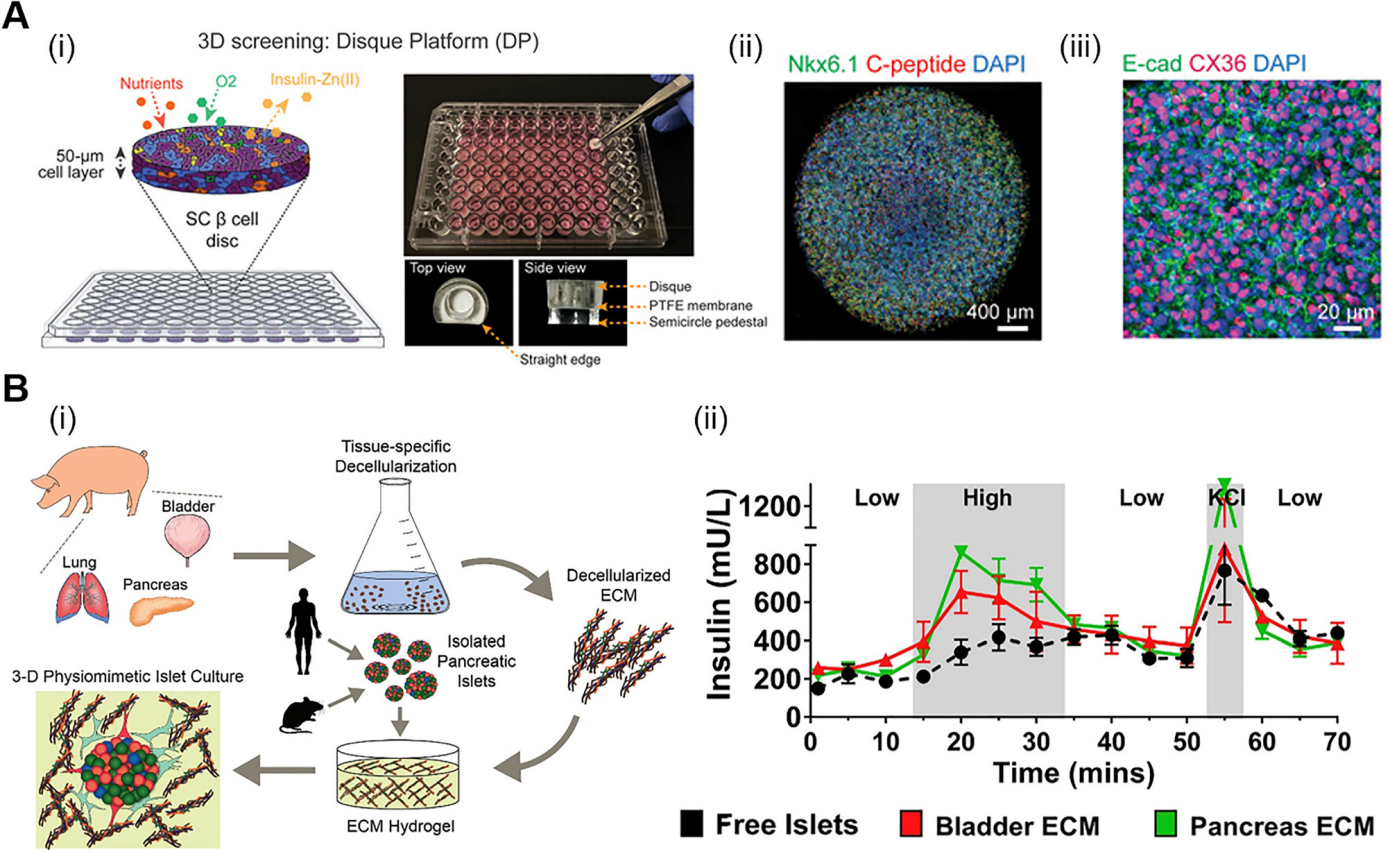
3D microenvironment and tissue-specific effects of ECM. (a) (i) Illustration of the 3D culture platform for stem cell (SC)-derived beta cells. (ii) Immunofluorescent staining of NKX6.1 and C-peptide in DP. (iii) Expression of junctional markers (E-cadherin and connexin 36) in DP. Reproduced with permission from Yang *et al.*, Sci. Adv. **6**, eabc3207 (2020). Copyright 2020 Authors, licensed under a Creative Commons Attribution (CC BY) license.^46^ (b) (i) Schematic to illustrate the overall process of decellularization and encapsulation of islets. (ii) Evaluation of glucose responsiveness in free islets, bladder ECM, and pancreas ECM using the glucose stimulated insulin release test. Reprinted with permission from Jiang *et al.*, Biomaterials **198**, 37–48, Copyright 2019 Elsevier.^53^

As a first step toward native tissue mimicry *in vitro*, recent studies have shown that encapsulation strategies can boost the islet-like cellular function of hiPSC-derived beta cells by facilitating cell-matrix interactions.^43–45^ Legøy *et al.* investigated whether encapsulation with 3D alginate hydrogel during the differentiation process could result in effective differentiation outcomes. Their global proteome analysis data showed that hierarchical clusters of encapsulated cells were closer to human islets than those of 2D differentiated cells, indicating that differentiation in the 3D microenvironment strongly promotes the fate of human pancreatic endocrine cell. More recently, Yang *et al.* applied stem cell-derived beta cells to the 3D culture system termed Disque Platform (DP) for screening zinc-binding prodrugs related to beta cell proliferation [Fig. 3(a)].^46^ The developed DP included core extracellular matrix (ECM) components such as laminin and collagen type IV that are involved in human islet development. Consequently, they observed higher expression rates of key transcription factors (e.g., PDX1 and NKX6.1) and junctional structures (e.g., E-cadherin and connexin 36) in the 3D reconstituted beta cells inside the DP compared to 2D monolayer conditions. In this study, they underlined the importance of reproducing the 3D pancreatic niche to improve cell-ECM interactions in fabricating a reliable drug testing platform for DM research.

The recapitulation of a 3D microenvironment can be designed with diverse biomaterials, among them, decellularized ECM (dECM) holds outstanding potential with respect to furnishing target tissue-specific niche to cells.^47–50^ This is because dECM preserves the tissue-specific ECM composition that can provide not only stable structural integrity but also abundant biochemical signals of native tissues and organs.^51^ Han *et al.* reported that dECM regulates the directed differential behaviors of multipotent stem cells in a tissue-specific context.^52^ They categorized upregulated gene sets in four different types of dECM (liver, heart, cornea, and skin dECM) using transcriptome analysis and demonstrated that the uniqueness of each dECM may contribute to efficient differentiation into a specific target lineage. Specifically, in the study by Bi *et al.*, iPSC-derived islet organoids that were differentiated on a collagen type V substrate, which had been exclusively identified in pancreas dECM via proteomic analysis, exhibited high expression levels of endocrine lineage related markers and the higher glucose responsiveness compared to that of Matrigel only group.^45^ Furthermore, Jiang *et al.* proved that pancreas dECM can facilitate long-term cell-matrix communication by reporting high islet viability after 80 days. Their glucose-stimulated insulin release test results suggested that the recapitulation of the peri-islet niche using pancreas ECM had a more positive effect on glucose sensitivity of the encapsulated islets than using other types of tissue-derived ECM [Fig. 3(b)].^53^ Taken together, these studies have emphasized the significance of tissue-specific identity and the beneficial effects of dECM in recreating the microenvironment of a desired organ *in vitro*.

### B. Modeling DM based on microfluidics-based organs-on-chips

The manipulation of biomimetic flow based on the microfluidic system can be a key strategy to engineer more-controllable environments.^54,55^ To be specific, microfluidic devices can provide cells with dynamic biomechanical signals through precisely controlled shear stress, enabling real-time interpretation of certain physiological phenomena.^14^ Many studies have demonstrated the fabrication of microfluidic devices for the purpose of assessing the function of isolated islets prior to transplantation.^56–60^ For example, islet-on-a-chip proposed by Glieberman *et al.* had three operating principles (e.g., insulin calibration to produce the standards, islet trapping, and glucose stimulation with high glucose media) for the continuous measurement of secreted insulin via synchronized glucose pulses. The development of a scalable chip to test islet potency through automated real-time readouts is, thus, a robust engineering approach that can accelerate DM research.

**FIG. 4.**
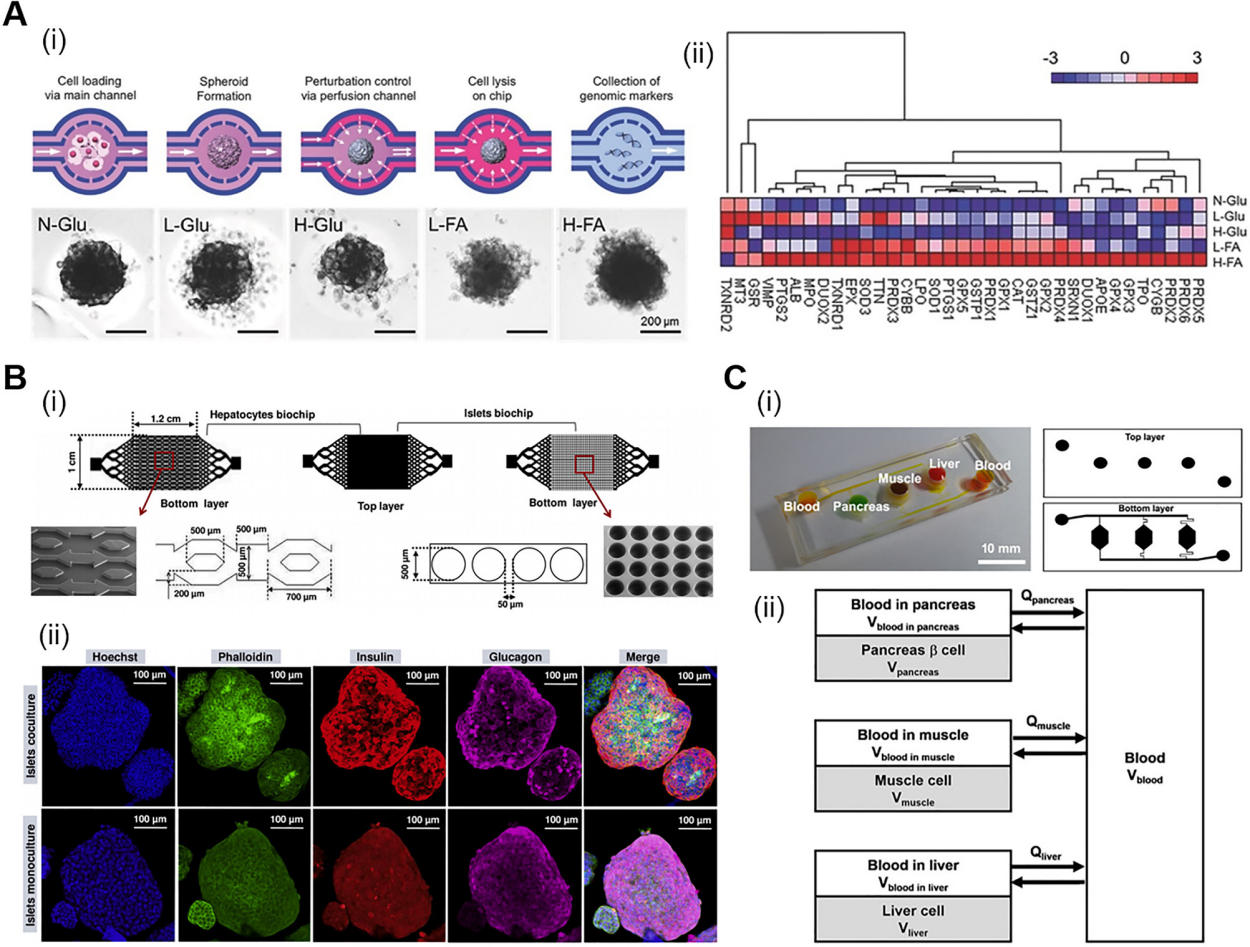
DM modeling on the microfluidic platform. (a) (i) Diabetic stimulation with glucose (Glu) and fatty acids (FA) to islet spheroids. (N-normal, L-low, and H-high). (ii) Upregulated gene sets related to oxidative stress pathway have largely shown in H-FA conditions (upregulated—red, downregulated—blue). Reproduced with permission from Lee *et al.*, Adv. Healthcare Mater. **7**, 1701111 (2018). Copyright 2018 John Wiley & Sons.^65^ (b) (i) Schematic of hepatocytes and islets chip designs for DM modeling. (ii) Immunofluorescent imaging of islets in monoculture and coculture conditions. Reprinted with permission from Essaouiba *et al.*, Biochem. Eng. J. **164**, 107783 (2020). Copyright 2020 Elsevier.^66^ (c) (i) Gross view of pancreas-muscle-liver MPS. (ii) Schematic of the mathematical model for the multi-organ MPS. Reproduced with permission from Lee *et al.*, Biotechnol. Bioeng. **116**, 3343–3445 (2019). Copyright 2019 John Wiley & Sons.^67^

Intriguingly, recent technological breakthroughs in organs-on-chips technology have extended the applicability of microfluidic devices to platforms that can simulate *in vivo* environments based on controlled fluid flow for disease modeling *in vitro*.^61–64^ For instance, Liu *et al.* created a compartmentalized co-culture model of adipocytes and immune cells using a fluidic chip system to study the immune-metabolic reactions that occur in patients with T2DM.^62^ Adipocytes co-cultured with immune cells revealed higher glucose uptake rates after insulin treatment than in the monoculture condition, proving that insulin resistance, a representative indicator of T2DM, can be observed under conditions of co-culture with immune cells. Moreover, Lee *et al.* generated a microphysiological analysis platform to observe the pathophysiological mechanisms of human pancreatic beta cells under diverse diabetic stimulations by controlling the concentration of glucose and fatty acids.^65^ They optimized the formation of beta cell spheroids using computational simulations of the flow profiles and then exposed the spheroids to glucolipotoxicity stimulus to analyze the genomic markers related to beta cell dysfunction. As a result, upregulated clusters of oxidative stress-responsive genes were found at high concentration of the palmitic acid condition. Their findings previewed the potential of microfluidic-based organ models in discovering specific pathways related to lipidemia in the human DM condition by combining with genetical research [Fig. 4(a)].

The organs-on-chips technology offers the advantage of modeling multi-organ pathologies that allows the observation of organ-to-organ interactions in one system connected through microfluidic channels. Essaouiba *et al.* established a pancreas-liver-on-a-chip system to illustrate the systemic crosstalk between the key organs affected by insulin resistance in DM development.^66^ The proposed pancreas-liver-on-a-chip system led to higher insulin secretion and the recovery of hepatic functions via glucose homeostasis; on the other hand, lower albumin production was observed for monocultured hepatocytes in the absence of insulin. Moreover, co-cultured pancreatic islets represented a high expression level of insulin compared to the monocultured islets [Fig. 4(b)]. Engineering multiple organs within one platform to reproduce the complex communication between the diabetic tissues can enable new physiomimetic DM modeling approaches.

In a recent study carried out by Lee *at al.*, an *in vitro* pancreas-liver-muscle model based on a microphysiological system (MPS) with a mathematical approach was constructed to capture the dynamic interactions between multiple organs in glucose metabolism [Fig. 4(c)**]**.^67^ They applied allometric scaling methods considering physiological variables between organisms with varying mass.^68,69^ Their model provided a methodological framework to determine the scaling for each organ module to simulate physiologically relevant metabolic activities, including glucose uptake and insulin secretion. Collectively, the mimicking of *in vivo* metabolic phenomena between multi-organs via mechanical inputs, such as shear flow, can be more accurately recapitulated using organs-on-chips systems. Furthermore, complementing the main organs affected by DM with vasculatures could further maximize pathological relevance in the chip-based DM modeling.

### C. Modeling DM using 3D bioprinting technology

Bioprinting has emerged as an advanced technology for building 3D DM models *in vitro*. Bioprinting allows the implementation of tissue-specific 3D geometries via the precise positioning of cells and printable inks.^42,47,48,70–73^ With respect to this strategy, Kim *et al.* selected skin tissue-derived dECM bioink formulated with human dermal fibroblasts (HDFs) and human epidermal keratinocytes (HEKs) to fabricate functional 3D skin tissue constructs.^74^ They induced the differentiation of diabetic HEKs using the cellular crosstalk effects between the printed diabetic HDFs-loaded dermal layer and the normal HEKs-loaded epidermal layer. The stratified epidermal layers were observed to be thinner than those under normal conditions, which correlates with the findings in the native epidermis of diabetic patients. In addition, a delay in the re-epithelization process with the engineered diabetic substrate was observed in the wounded skin model, which was constructed using a printing technique. This study presents a physiomimetic skin model for clinical DM studies that can mimic not only structural characteristics in actual skin but also pathophysiological responses that cause diabetic foot ulcer.

**FIG. 5.**
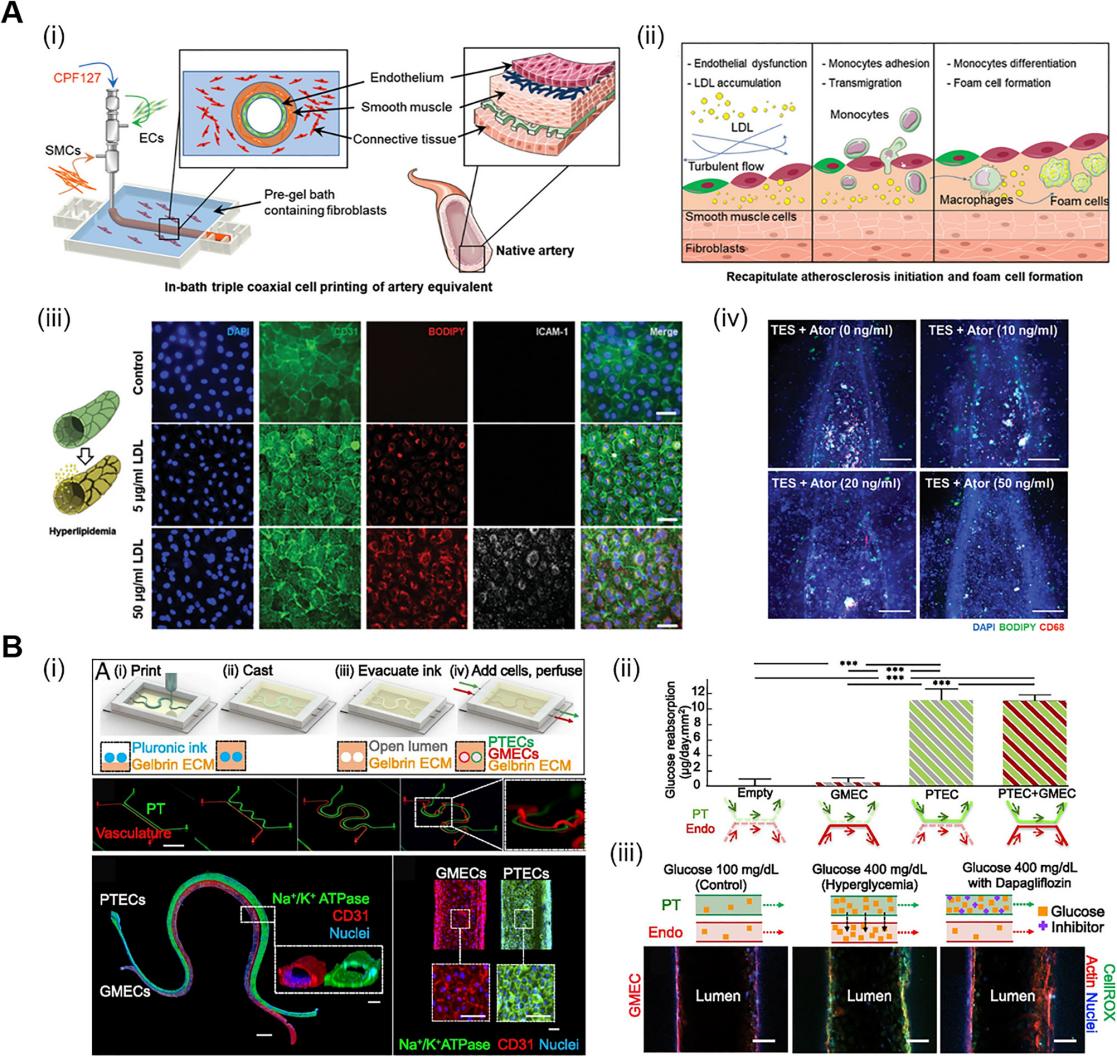
Modeling DM complications using 3D bioprinting technologies. (a) (i) Schematic illustration of the AE model using the 3D coaxial cell printing technique. (ii) Schematic diagram of key events involved in atherosclerosis progression. (iii) Expression of CD31, BODIPY, and ICAM-1 at the high concentration of the LDL treated condition. Scale: 50 *μ*m. (iv) Evaluation of foam cell formation after atorvastatin treatment (TES-turbulent-flow model, which contains EC, smooth muscle cells, and fibroblasts). Scale: 200 *μ*m. Reproduced with permission from Gao *et al.*, Adv. Funct. Mater. **31**, 2008878 (2020). Copyright 2020 John Wiley & Sons.^75^ (b) (i) Fabrication process of the 3D VasPT model. Scale: 10 mm. (ii) Quantification of glucose reabsorption in four different conditions. Error bars: SD of the mean, *p < 0.05; **p < 0.001; ***p < 0.0001. (iii) Experimental conditions of hyperglycemia and administration of dapagliflozin in the 3D bioprinted PT model. Scale: 100 *μ*m. Reproduced with permission from Lin *et al.*, Proc. Natl. Acad. Sci. U.S.A. **116**, 5399–5404, 2019. Copyright 2019, Authors, licensed under a Creative Commons Attribution (CC BY) license.^79^

As mentioned above, diabetic complications are mainly caused by damage to the vasculatures due to hyperlipidemia. For mimicking DM with vascular complications, implementation of the *in vivo*-like vascular structure is essentially required. Among the various bioprinting systems (e.g., ink-jet printing and laser-assisted printing), extrusion-based printing strategies hold huge potential in vascular disease modeling.^73,75–80^ Specifically, the coaxial printing technique, one of the micro-extrusion printing methods, is specialized in mimicking the vessel architecture *in vitro* by locating the endothelium layer in core with sacrificial inks and the smooth muscle layer in shell to create a 3D tubular structure. Gao *et al.* developed an atherosclerosis model based on in-bath coaxial cell printing that recapitulated the cellular and geometrical configurations of mature vascular tissue with a triple-layered artery equivalent (AE) **[**Fig. 5(a)**]**.^75^ Reflecting the excellent performance of vascular tissue-derived dECM (VdECM) bioink reported in the previous study, they further demonstrated the additional potential of VdECM bioink as a bath material capable of supporting a stable printed vessel structure *in vitro*. They characterized the dysfunction of vascular tissue after the induction of hyperlipidemia by perfusing low-density lipoprotein (LDL) into the AE model. The evident expression of ICAM-1, which is related to migration to the site of inflammation, was observed in the high concentration of LDL treated group. They employed turbulence flow and immune cells in the printed AE model to recapitulate key manifestations in atherosclerosis and then tested the effects of atorvastatin, a medication generally prescribed to reduce LDL accumulation in T2DM patients. Decreased number of foam cells, formed due to atherosclerosis, was observed in the printed model, and the dose-dependent effects of atorvastatin were also confirmed. This printed vascular model, which comprises connective tissue, smooth muscles, and endothelium to recapitulate native layers of human vessels, can contribute to the advancement of generating realistic organs *in vitro*.

Another interesting study by Lin *et al.* simulated the DM conditions related to diabetic nephropathy using a 3D vascularized proximal tubule (3D VasPT) model fabricated with a bioprinting approach [Fig. 5(b)].^79^ They reported the importance of the colocalization of the vasculature and proximal tubules in the 3D VasPT model for recapitulating the reabsorption of glucose, a key phenomenon in native kidney tissue. Two separate tubular structures positioned adjacent to each other on the platform; one was seeded with proximal tubule epithelial cells (PTECs) and the other with glomerular microvascular endothelial cells (GMECs). This setup produced cells with a mature phenotype and high glucose reabsorption efficiency via the active crosstalk. To investigate anti-diabetic drug responses using the 3D VasPT model, hyperglycemia was induced by circulating a high concentration of glucose into the tubules. Damaged junction structures and increased intensity of CellROX and nitrotyrosin, oxidative stress makers, were observed in the GMECs under hyperglycemic conditions. Finally, dapagliflozin, which can protect ECs from excessive glucose transport via the inhibition of the glucose transporter located on the renal cells, was used to treat the diseased 3D VasPT model. As a result, the restoration of endothelial function was observed with the significantly decreased expression of the oxidative stress markers in the GMECs. Such models can enhance our understanding of the diverse pathomechanisms of DM. Furthermore, applying diabetic patient-derived cell sources to bioprinting technology will enable the creation of more physiologically relevant symptoms in a patient-specific manner.

Many studies have verified that bioprinting technology is a suitable method for the fabrication of scalable and modular tissue analog via patterning and layering of the desired tissue.^70,71,81–85^ Moreover, bioprinting allows the development of the organs-on-chips system via the outlining housing system using biomaterial inks and positioning various bioinks at the same time in an automated manufacturing process.^86^ After connecting a micropump to the bioprinted organs-on-chips system, a high quality *in vitro* human body platform can be generated with *in vivo*-like interstitial flow, enabling disease modeling and the observation of physiological changes, which are the key advantages of organs-on-chips technology. Taken together, bioprinting technology offers a versatile biofabrication strategy for the development of functional *in vitro* DM models that can meet tissue-specific design criteria through effective organ manufacturing.

## IV. GENERAL CHALLENGES FOR MODELING DM *IN VITRO*

A key challenge in creating DM models is achieving the functional maturation of *in vitro* tissue models by recapitulating the biological complexity of native tissue. Despite continued efforts to develop protocols to fully differentiate mature target cells, the gradual loss of cell functionality *in vitro* remains an issue.^87–90^ In the native pancreatic islets, vascular cells not only deliver nutrients and oxygen to beta cells but also enhance insulin transcription and secretion via endothelium-derived factors (e.g., connective tissue growth factors, hepatocyte growth factors, thrombospondin-1, fibroblast growth factor, and vasoconstrictive endothelin-1).^91,92^ Beta cells secrete vascular endothelial growth factor-A, which is important for the maintenance of proper density and the functional structure of the intra-islet vasculatures.^93^ Thus, the recapitulation of the cellular microenvironment with vascular networks inducing bidirectional interactions between target cells and ECs will be a useful strategy for overcoming the limited maturation of *in vitro* tissue models. Additionally, human islet stromal cells, which surround dense clusters of metabolic cells, secrete pancreatic ECM constituents (e.g., collagen type I, IV, and VI, fibronectin, and laminin) and express a mesenchymal stromal cell-like phenotype.^94,95^ In this regard, recent advances in pancreatic tissue engineering have strongly suggested that intrinsic vascularization using co-culture strategies with supporting cells may lead to the maturation of beta cells *in vitro*.^96–106^ Takahashi *et al.* reported that a co-culture of human umbilical vein endothelial cells (HUVECs) and mesenchymal stem cells (MSCs) with iPSCs produces self-condensed tissue, which can be universally adapted to a diverse range of tissue fragments, and represents an effective endothelialization method.^106^ Similarly, Yoshihara *et al.* generated multicellular spheroids (MCSs) by introducing human adipose-derived stem cells, which resemble pancreatic fibroblasts and HUVECs to the process of differentiation into beta cells. The MCSs had a higher expression level of metabolic maturation markers such as ESRRG and better insulin secretion ability than did the beta cell only group.^103^

Improvements of further niche by adding vessel-specific ECM components can be a beneficial strategy for vascularization *in vitro*. Gao *et al.* fabricated hybrid bioinks composed of alginate and VdECM to print vessel constructs.^76^ They assessed the angiogenesis observed in vascular cells encapsulated in hybrid bioinks by comparing it with other conventional bioinks such as collagen and alginate. Interconnected vessel-like structures and increased gene expression (e.g., CD31, VE-cadherin, and vWF) in the endothelial progenitor cells were observed with hybrid bioinks but not with the other types of bioink. Another strategy for vascularization that leads to maturation is external stimuli with dynamic flow. Jun *et al.* characterized the shear-activated expansion of islet ECs on their platform and compared this to a static environment.^107^ They explained that, under an optimal flow rate, the islets in the dynamic situation form a tightly organized architecture with abundant microvilli on the surface, which contain signaling microdomains for insulin secretion. Furthermore, controlling the angiogenic sprouting behavior of vascular cells via spatial positioning based on bioprinting technology is a promising approach for improving vascularization *in vitro*.^108,109^ Jeon *et al.* attempted to optimize micro-patterns by controlling the distance between hepatic spheroids and ECs using 3D bio-dot printing. As a result, a significant enhancement in the metabolic functionality of hepatocytes was observed when the hepatic spheroids were positioned far from the ECs. Altogether, the organization of the functional vasculature by optimizing the cellular composition and the addition of target tissue-specific ECM components, biomimetic flow, and 3D topographical cues with precise patterning can lead to the maturation of 3D *in vitro* tissue platforms for DM modeling.

## V. CONCLUSION AND FUTURE DIRECTIONS

Technical advances in therapeutic options for patients with T1DM have enabled continuous insulin delivery using automated artificial pancreas systems rather than daily exogenous insulin injections. However, it is difficult to mimic physiological responses in the islet, gut, and liver, which are critical organs for glycemic control, through the replacement of insulin via a subcutaneous artificial pancreas.^87^ For this reason, the reversal of diabetes via functional islet transplantation that can recapitulate the role of endogenous beta cells has emerged as a pressing goal.^110,111^ In addition, further efforts to overcome the limited supply of cadaveric islets have led to aggressive investigations into the generation of stem cell-derived beta cells *in vitro*.^100,112,113^ However, in-depth research maintaining the long-term functionality of differentiated cells for the therapeutic regeneration of beta cells is still required. For T2DM, pharmacotherapies based on a patient's level of glycated hemoglobin and the strict management of their lifestyle, such as diet and exercise, are common treatments.^114^ In particular, the American Diabetes Association strongly recommends individualized therapy guided by glycemic efficiency and consideration of the risk of hypoglycemia. In this respect, in order to minimize the side effects of pharmaceuticals, intensive research on the action mechanisms of drugs based on the pathophysiology of individual patients is desperately needed. A safer and more potent therapeutic approach using a personalized *in vitro* tissue platform would be able to reduce the socioeconomic burden of DM pathologies.

This review elucidates that 3D *in vitro* tissue platforms based on a diverse range of engineering approaches have significant potential in developing faithful platforms to study DM. A recent study designed to create functionally mature beta cells suggested the manufacture of clinically applicable cell sources that meet Good Manufacturing Practice standards for the prediction of realistic clinical outcomes.^104^ Along the same lines, MSCs, for which thousands of clinical trials have been registered at FDA.gov, may be a suitable candidate for use as clinically relevant functional cells for *in vitro* tissue.^115^ For *in vivo* recapitulation, reconstruction of dECM to further optimize 3D tissue-specific niches can be achieved by modifying the compositional ratio of ECM proteins after a comparison of native tissue with decellularized tissue via proteomic analysis.^116^ Referring to the comprehensive human tissue proteome studies, the supplementation of relatively deficient ECM proteins in the decellularized tissue compared to the native tissue will improve tissue-specific cues *in vitro*.^117^ Furthermore, incorporation of perfusion and cell patterning can bring potent synergetic effects for the generation of advanced DM models by mimicking blood flow and reproducing complex geometrical features of the human body. Recently, a novel approach based on electrical engineering illustrated the possibility of utilizing digital input to directly control the cellular behavior of human beta cells.^118^ Overall, next generation of *in vitro* DM models can be fabricated with such a variety of engineering possibilities.

## Data Availability

The data that support the findings of this study are available from the corresponding author upon reasonable request.
